# Differential Effects of Intra-Abdominal Hypertension and ARDS on Respiratory Mechanics in a Porcine Model

**DOI:** 10.3390/medicina60060843

**Published:** 2024-05-22

**Authors:** Benjamin Seybold, Anna M. Deutsch, Barbara Luise Deutsch, Emilis Simeliunas, Markus A. Weigand, Mascha O. Fiedler-Kalenka, Armin Kalenka

**Affiliations:** 1 Department of Anesthesiology, Medical Faculty, Heidelberg University Hospital, University Heidelberg, 69120 Heidelberg, Germany; annamagdalena.deutsch@vivantes.de (A.M.D.); luisedeutsch@gmx.de (B.L.D.); simeliunui@gmail.com (E.S.); markus.weigand@med.uni-heidelberg.de (M.A.W.); mascha.fiedler-kalenka@med.uni-heidelberg.de (M.O.F.-K.); armin.kalenka@med.uni-heidelberg.de (A.K.); 2 Department of Anesthesiology, Intensive Care Medicine and Pain Therapy, Vivantes Klinikum im Friedrichshain, 10249 Berlin, Germany; 3 Department of Anesthesiology, Intensive Care and Emergency Medicine, Asklepios Klinik Wandsbek, 22043 Hamburg, Germany; 4 Department of Anesthesiology and Intensive Care Medicine, Bürgerspital Solothurn, 4500 Solothurn, Switzerland; 5 German Center for Lung Research (DZL), Translational Lung Research Center Heidelberg (TLRC), 69120 Heidelberg, Germany; 6 Hospital Bergstrasse, 64646 Heppenheim, Germany

**Keywords:** respiratory mechanics, acute respiratory distress syndrome, intra-abdominal hypertension, transpulmonary pressure, strain, compliance, elastance

## Abstract

*Background and Objectives*: Intra-abdominal hypertension (IAH) and acute respiratory distress syndrome (ARDS) are common concerns in intensive care unit patients with acute respiratory failure (ARF). Although both conditions lead to impairment of global respiratory parameters, their underlying mechanisms differ substantially. Therefore, a separate assessment of the different respiratory compartments should reveal differences in respiratory mechanics. *Materials and Methods*: We prospectively investigated alterations in lung and chest wall mechanics in 18 mechanically ventilated pigs exposed to varying levels of intra-abdominal pressures (IAP) and ARDS. The animals were divided into three groups: group A (IAP 10 mmHg, no ARDS), B (IAP 20 mmHg, no ARDS), and C (IAP 10 mmHg, with ARDS). Following induction of IAP (by inflating an intra-abdominal balloon) and ARDS (by saline lung lavage and injurious ventilation), respiratory mechanics were monitored for six hours. Statistical analysis was performed using one-way ANOVA to compare the alterations within each group. *Results*: After six hours of ventilation, end-expiratory lung volume (EELV) decreased across all groups, while airway and thoracic pressures increased. Significant differences were noted between group (B) and (C) regarding alterations in transpulmonary pressure (TPP) (2.7 ± 0.6 vs. 11.3 ± 2.1 cmH_2_O, *p* < 0.001), elastance of the lung (E_L_) (8.9 ± 1.9 vs. 29.9 ± 5.9 cmH_2_O/mL, *p* = 0.003), and elastance of the chest wall (E_CW_) (32.8 ± 3.2 vs. 4.4 ± 1.8 cmH_2_O/mL, *p* < 0.001). However, global respiratory parameters such as EELV/kg bodyweight (−6.1 ± 1.3 vs. −11.0 ± 2.5 mL/kg), driving pressure (12.5 ± 0.9 vs. 13.2 ± 2.3 cmH_2_O), and compliance of the respiratory system (−21.7 ± 2.8 vs. −19.5 ± 3.4 mL/cmH_2_O) did not show significant differences among the groups. *Conclusions*: Separate measurements of lung and chest wall mechanics in pigs with IAH or ARDS reveals significant differences in TPP, E_L_, and E_CW_, whereas global respiratory parameters do not differ significantly. Therefore, assessing the compartments of the respiratory system separately could aid in identifying the underlying cause of ARF.

## 1. Introduction

Acute respiratory failure (ARF) is a prevalent cause of intensive care unit (ICU) admission [1]. Despite the inherent risk of ventilator-induced lung injuries (VILI), invasive mechanical ventilation remains a vital intervention for patients with ARF [2,3]. Efforts to understand the physics of mechanical ventilation have intensified in recent decades, aiming to minimize potential harm and optimize the benefits of positive-pressure ventilation [3].

ARF may be caused by primarily intrapulmonary issues, such as pneumonia, leading to so-called primary acute respiratory distress syndrome (ARDS) in several patients. ARDS, which still causes high mortality today, affects about 10% of ICU patients [1,4]. Characteristic findings in patients with ARDS include a reduction in functional residual capacity (FRC) and elevated airway pressures during mechanical ventilation [5,6]. Common extrapulmonary factors, such as intra-abdominal hypertension (IAH), prevalent among ICU patients, can also induce secondary respiratory complications, leading to ARF [7,8,9]. In a supine position, roughly half of intra-abdominal pressure transmits to the intrathoracic compartment [7,10,11]. Therefore, IAH has a direct impact on the thoracic cavity, resulting again in a reduction of FRC and elevated airway pressures [12].

Initially, the physical interactions between the abdominal and thoracic compartments may seem obvious but are challenging to identify and to handle in clinical practice. Severe impairment of global respiratory parameters and an increase of mechanical load of the lungs have been found in patients with ARF due to IAH and primary ARDS, despite different components of the respiratory system being primarily affected [3,8,11,13,14]. Regarding respiratory mechanics, these different components include the lung and the chest wall [8].

Since treatment strategies differ substantially based on ARF etiology (e.g., abdominal decompression or prone positioning), identifying the primary problem is imperative [4,14,15,16,17]. Therefore, we investigated lung and chest wall mechanics in a porcine model of artificially induced IAH and acute primary lung injury. We aimed to descriptively demonstrate in an experimental animal trial that lung and chest wall mechanics differ between the groups, while global respiratory parameters, routinely measured in the ICU, do not. Our findings could help identify the major problems of patients with ARF through minimal-invasive measurements, enabling the selection of appropriate treatment and ventilator settings to improve patients’ outcomes.

## 2. Materials and Methods

### 2.1. Study Settings

This study was a prospective, experimental trial involving 18 female domestic pigs. Some data from our lab have been partially published before [18,19]. The pigs were divided into three groups (A, B, and C), each comprising six animals. Group A (control group), with an intra-abdominal pressure (IAP) of 10 mmHg and no ARDS, served as the comparison group, representing an average IAP in ICU patients [20]. Group B (IAH group), with an IAP of 20 mmHg and no ARDS, represented an experimental model of intra-abdominal hypertension. Group C (ARDS group), with an IAP of 10 mmHg and an artificially induced primary lung injury, represented an experimental model of early ARDS [21].

### 2.2. Ethics and Registry

The protocol received approval from the relevant animal research committee (Regierungspräsidium Karlsruhe, No. 35-9185.81/G-161/17). All animals were housed in the interfaculty biomedical facility of the University of Heidelberg and were sourced from a local pig breeder. All procedures were conducted in accordance with the animal welfare regulations stipulated by German law.

### 2.3. Animal Preparation

After overnight fasting with free access to water, the pigs were intramuscularly anesthetized with the following doses: 7 mg/kg Azaperon (Stresnil, Lilly, Bad Homburg, Germany), 8 mg/kg Ketaminhydrochlorid (Ketamin 10%, Bremer Pharma, Warburg, Germany), and 0.3 mg/kg Midazolam (Midazolam, Hameln Pharma, Hameln, Germany). Anesthesia was maintained through continuous infusion of 6 mg/kg/h Esketamin (Ketanest S, Pfizer Pharma, Berlin, Germany), 3.6 mg/kg/h Midazolam, and 10–30 mg/kg/h Propofol (Propofol 2%, Fresenius Kabi, Bad Homburg, Germany). No neuromuscular blockers were used. The adequacy of anesthesia depth was regularly assessed by monitoring for the absence of spontaneous breathing efforts and muscle tone. A continuous infusion of crystalloid fluids (Sterofundin^®^, Braun, Melsungen, Germany) at a rate of 10 mL/kg/h was administered during the first hour. Subsequently, the infusion rate was adjusted to 10–40 mL/kg/h to maintain hemodynamic stability. Catecholamines (noradrenaline) were administered sporadically in group C during saline lung lavage to maintain stable hemodynamics. Throughout the experimental period, no further administration of catecholamines was required in any of the groups.

The pigs were maintained in supine position throughout the experiment. After anesthesia induction, the animals were tracheotomized and mechanically ventilated using an intensive care ventilator (Carescape R860, GE Healthcare, Madison, WI, USA) in a pressure-controlled mode with volume guaranty. The initial ventilator settings were as follows: inspiratory oxygen concentration (FiO_2_) 0.4, tidal volume (V_t_) of 8 mL/kg body weight, inspiratory-to-expiratory ratio (I:E) of 1:2, respiratory rate (RR) of 20 breaths per minute, and positive end-expiratory pressure (PEEP) of 5 cmH_2_O. No recruitment maneuvers were applied.

A central venous catheter (Logicath, Smith medical, Grasbrunn, Germany) was placed in the external jugular vein, and an arterial thermistor-tipped catheter (PiCCO^®^, Pulsation medical systems, Feldkirchen, Germany) was inserted into a femoral artery using ultra sound guidance (VScan^®^, GE ultrasound, Horten, Norway). A polyethylene catheter with an esophageal pressure probe (Nutrivent multifunction nasogastric catheter, Sidam, San Glacomo Roncole, Italy) was orally inserted into the stomach and connected to the ventilator for esophageal pressure (PEs) measurement. Proper installation and positioning were verified as previously described [22]. Additionally, a urinary catheter (UnoMeter^®^ Abdo-Pressure, ConvaTec, Birkerod, Denmark) with a pressure probe for intrabdominal pressure measurement was placed into the bladder.

A large balloon (200-liter weather balloon, Stratoflight, Blomber, Germany) was placed into the peritoneal cavity following midline laparotomy. Proper positioning in all abdominal quadrants was ensured through visual inspection and partial inflation. Subsequently, the abdomen was carefully closed. After baseline measurement, intra-abdominal pressure was adjusted by inflating the balloon with water.

Acute lung injury was induced in the ARDS group as previously described by repeatedly instilling of 0.9% sodium chloride into the endotracheal tube until a ratio of partial arterial pressure of oxygen to inspired oxygen (P/F ratio) <150 mmHg was reached for at least 30 min [23]. Following this procedure, injurious mechanical ventilation was administered for 120 min using pressure-controlled mode with inspiratory airway pressure (P_insp_) set at 35 cmH_2_O, PEEP at 0 cmH_2_O, and a respiratory rate of 12/min, in order to induce primary lung injury representing an ARDS model [21]. As a representative model for early ARDS, primarily atelectasis is expected within the first six hours as a consequence of lung injury.

### 2.4. Measurements and Calculations

P_insp_, mean airway pressure (P_mean_), and PEEP, inspiratory and expiratory esophageal pressure (P_Esinsp_, P_Esexp_, respectively), were recorded from the ventilator. Transpulmonary inspiratory pressure (TPP_insp_) was calculated as TPP_insp_ = P_insp_ − P_Esinsp_, and transpulmonary expiratory pressure (TPP_exp_) as TPP_exp_ = PEEP − P_Esexp_. Airway driving pressure (ΔP) and transpulmonary pressure (TPP) were calculated as previously described [24]. Static compliance of the respiratory system (C_RS_ stat) and plateau airway pressure (P_plat_) were measured by the ventilator during an inspiratory hold maneuver. Static elastance of the respiratory system (E_RS_) was calculated as E_RS_ = (P_insp_ − PEEP)/V_t_, static chest wall elastance (E_CW_) as E_CW_ = (P_Esinsp_ − P_Esexp_)/V_t_ and static elastance of the lung (E_L_) as E_L_= E_RS_ − E_CW_. End-expiratory lung volume (EELV) was measured at the bedside as previously described [25] without interrupting mechanical ventilation on the designated PEEP level. Strain was calculated as strain = V_t_/EELV. Mechanical power (MP) was calculated as MP = 0.098 × V_t_ × RR × (P_plat_ − 0.5 × ΔP).

Heart rate, mean arterial pressure (MAP), heart index (HI), global end-diastolic volume index (GEDI), and extravascular lung water index (ELWI) were calculated with the PiCCO^®^ System. Settings of the PiCCO^®^ System were adjusted as recommended for the porcine model [26]. End-expiratory IAP was measured as recommended [27,28] and zeroed at midaxillary level [29].

Partial arterial pressure of oxygen (paO_2_), partial arterial pressure of carbon dioxide (paCO_2_), and lactate were measured with arterial blood gas (ABG) analysis. P/F ratio was calculated based on the ratio of paO_2_ to FiO_2_.

### 2.5. Experimental Protocol

After initial animal preparation as described above, the pigs were stabilized for 30 min before baseline (H0) data were recorded. Primary lung injury was induced only in group C by saline lung lavage and 120 min of injurious mechanical ventilation. The intra-abdominal balloon was then filled with water to achieve an IAP of 10 mmHg (groups A and C) and 20 mmHg (group B), respectively. PEEP was raised to 10 cmH_2_O in all groups. If necessary, FiO_2_ and RR were adjusted to maintain physiological conditions controlled by ABG.

**Figure 1:**
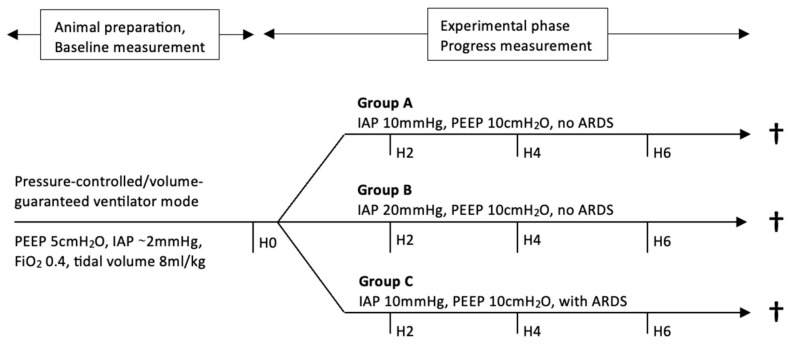
Timeline of the experimental protocol. †: euthanasia, ARDS: acute respiratory distress syndrome, cmH_2_O: centimeter of water, FiO_2_: inspiratory oxygen fraction, H: hour, IAP: intraabdominal pressure, kg: kilogram, ml: milliliter, mmHg: millimeter mercury, PEEP: positive end-expiratory pressure.

The subsequent experimental phase lasted another 6 h during which respiratory and hemodynamic data were recorded (see Figure 1). At the end of the experimental protocol, the pigs were euthanized with an intravenous bolus of 200 mg Propofol followed by 40 mmol potassium chloride.

Figure 1 illustrates the timeline (in hours) of the experimental protocol. Baseline parameters were assessed following animal preparation, prior to intaabdominal balloon inflation and ARDS induction (H0). Throughout the experimental phase, respiratory and hemodynamic parameters were recorded after two, four and six hours, respectively. Animals were euthanized at he end of the experiment.

### 2.6. Statistical Analysis

Sample size was calculated based on expected alterations in EELV from data from previous studies [18,19] and unpublished data in our lab.

Statistical analysis was performed using Microsoft Excel 16.80 (Microsoft Corporation, Redmond, WA, USA) and IBM SPSS 27 (IBM Corporation, Armonk, NY, USA, Version 27). Data are expressed as mean value ± standard error of the mean (SEM). Baseline values were analyzed using a one-way ANOVA. To compare the alterations (Δ) within the groups (baseline (H0) with the end of the experiment (H6)), we performed a one-way ANOVA followed by post hoc Bonferroni testing to determine specific pairwise differences between group means. The graphical representation of data was performed using GraphPad Prism 9 (GraphPad Software, San Diego, CA, USA).

## 3. Results

### 3.1. Baseline

We had three groups of six animals each: Group A (IAP 10 mmHg, no ARDS), Group B (IAP 20 mmHg, no ARDS), and Group C (IAP 10 mmHg, with ARDS). At the beginning of the experiment (baseline/H0), significant differences were observed between the mean values of the groups in body weight (49 ± 1; 37 ± 1; 49 ± 3 kg; *p* < 0.001), P_mean_ (8.5 ± 0.2; 8.3 ± 0.2; 9.3 ± 0.3 cmH_2_O; *p* = 0.036), P/F ratio (425 ± 20; 365 ± 15; 453 ± 18 mmHg; *p* = 0.009), heart rate (113 ± 9; 77 ± 10; 66 ± 7 bpm; *p* = 0.005), and mechanical power (8.9 ± 0.3; 6.3 ± 0.4; 9.3 ± 0.5 J/min; *p* < 0.001) (see Table 1).

### 3.2. Alterations after 6 h

All animals survived the experimental phase. Intra-abdominal pressures could be adequately adjusted by filling the balloons (see Table 2). Ventilator settings throughout the experimental phase were: Vt = 8 mL/kg body weight, PEEP = 10, I:E = 1:2.

### 3.3. Respiratory Mechanics after 6 h

**Figure 2:**
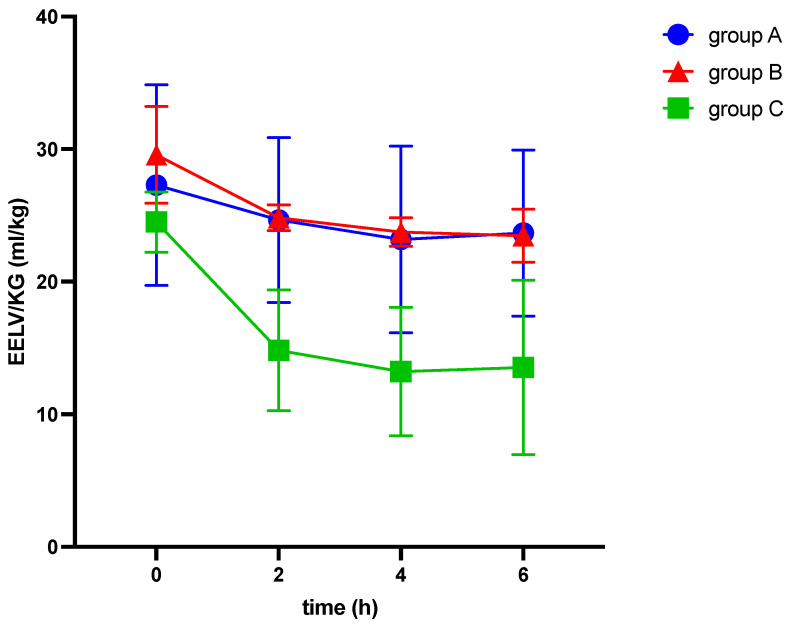
Alterations of EELV/kg bodyweight over six hours of ventilation. EELV: end-expiratory lung volume, h: hours, KG: kilogram bodyweight, kg: kilogram, ml: milliliter.

**Figure 3:**
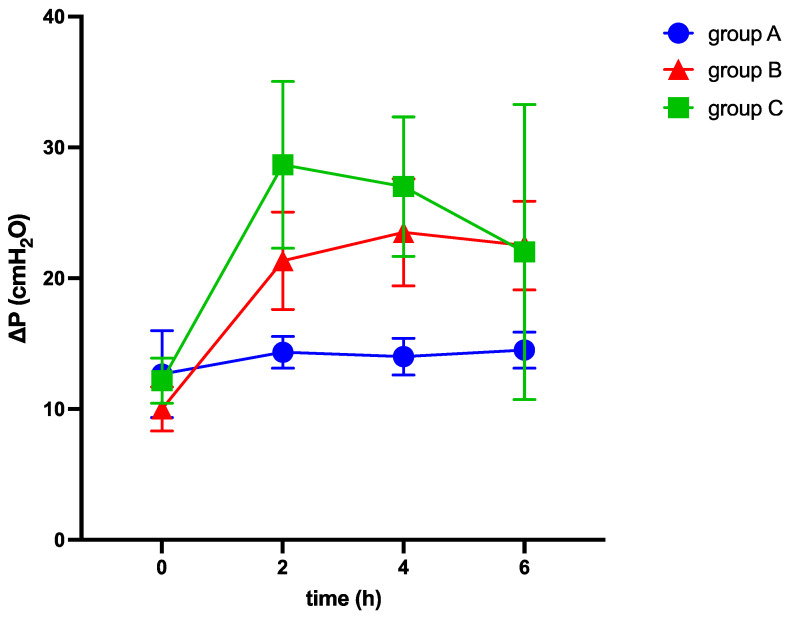
Alterations of driving pressure (ΔP) over six hours of ventilation. ΔP: driving pressure, cmH_2_O: centimeter of water, h: hours.

**Figure 4:**
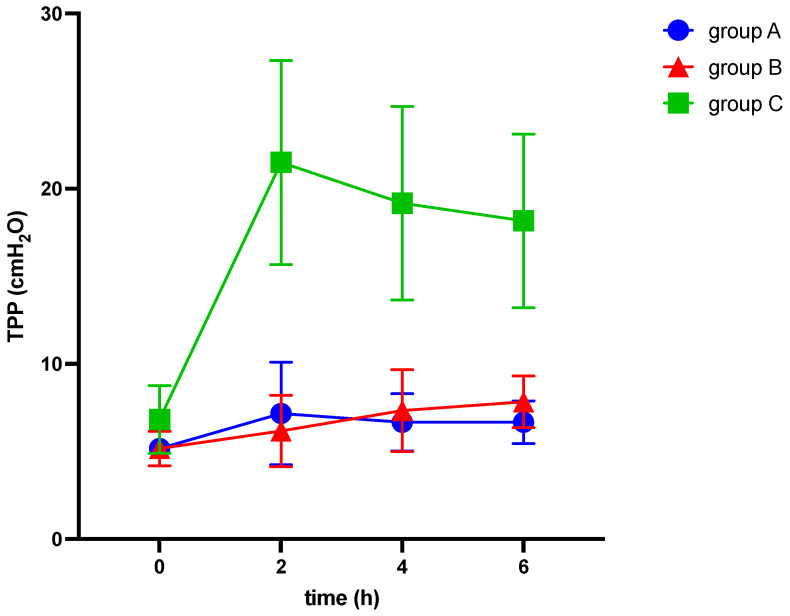
Alterations of transpulmonary pressure (TPP) over six hours of ventilation. cmH_2_O: centimeter of water, h: hours, TPP: transpulmonary pressure.

After 6 h, end-expiratory lunge volume decreased in all groups (see Table 2 and Figure 2), while airway and thoracic pressures were elevated (see Table 2 and Figure 3 and Figure 4).

Due to significant differences among the groups at baseline, we statistically compared the mean values of the alterations within the groups after six hours (ΔH6-H0).

Among other findings (see Table 2), we observed significant differences between the groups in various parameters, including EELV/kg (−3.6 ± 0.7; −6.1 ± 1.3; −11.0 ± 2.5 mL/kg; *p* = 0.022), TPP (1.5 ± 0.5; 2.7 ± 0.6; 11.3 ± 2.1 cmH_2_O, *p* < 0.001), ΔP (3.3 ± 0.6; 12.5 ± 0.9; 13.2 ± 2.3 cmH_2_O; *p* < 0.001), C_RS_ (−9.7 ± 2.7; −21.7 ± 2.8; −19.5 ± 3.4 mL/cmH_2_O; *p* = 0.027), E_RS_ (8.5 ± 1.6; 41.7 ± 3.1; 34.3 ± 6.5 cmH_2_O/mL; *p* < 0.001), and mechanical power (5.6 ± 0.3; 7.3 ± 0.3; 9.5 ± 0.9 J/min; *p* < 0.001) (see Table 2 and Figure 2, Figure 3 and Figure 4). The alteration of strain was substantially higher in group C than in both other groups, although not significantly different.

**Figure 5:**
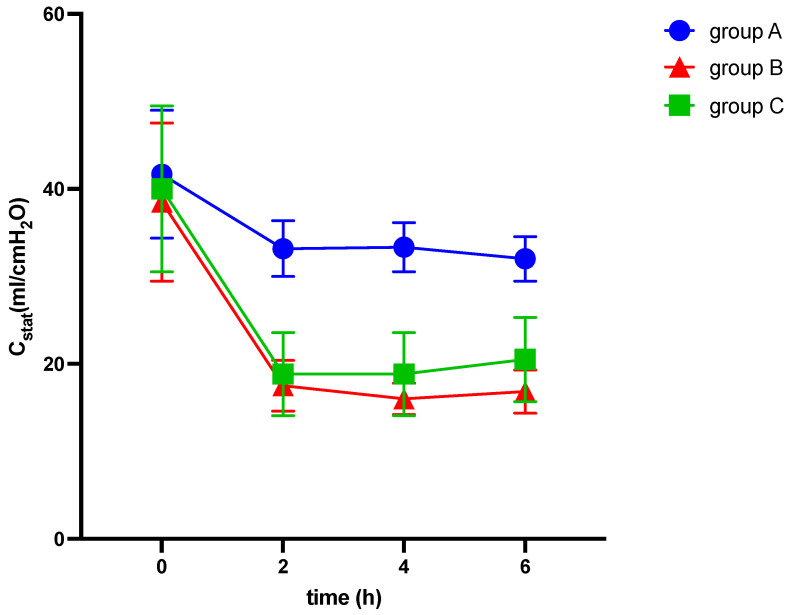
Alterations of compliance of the respiratory system (C_stat_) over six hours of ventilation. cmH_2_O: centimeter of water, C_stat_: static compliance of the respiratory system, h: hours, ml: milliliter.

**Figure 6:**
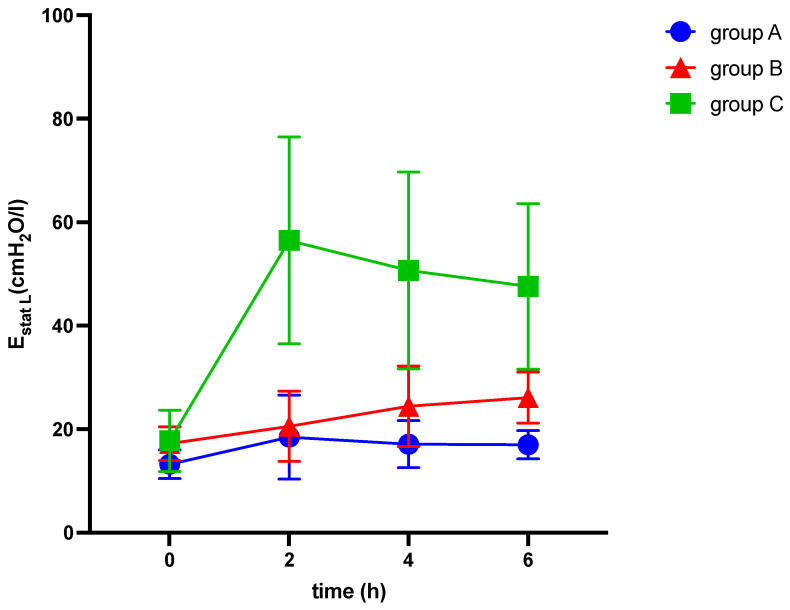
Alterations of elastance of the lung (E_stat L_) over six hours of ventilation. cmH_2_O: centimeter of water, E_stat L_: static elastance of the lung, h: hours, l: liter.

**Figure 7:**
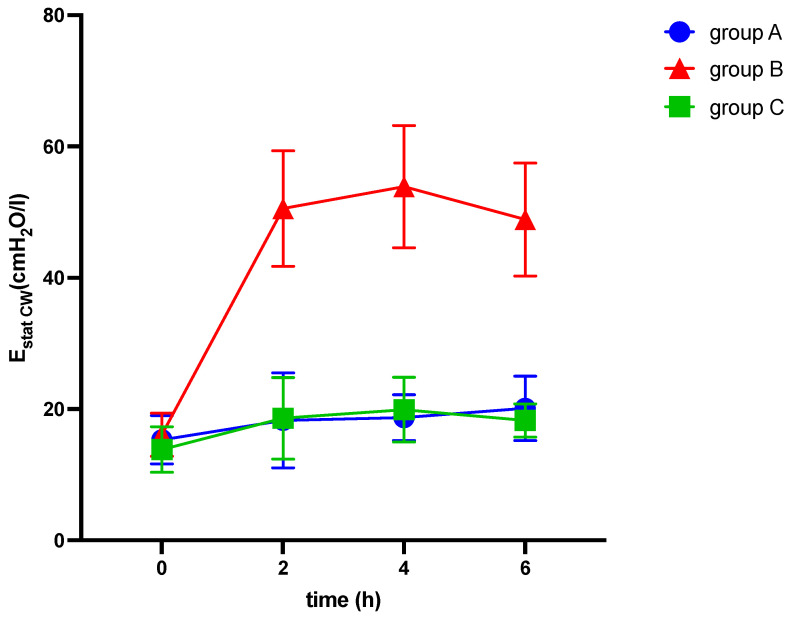
Alterations of elastance of the chest wall (E_stat CW_) over six hours of ventilation. cmH_2_O: centimeter of water, E_stat CW_: static elastance of the chest wall, h: hours, l: liter.

We observed significant differences between the IAH group and ARDS group in the alterations of TPP (2.7 ± 0.6 vs. 11.3 ± 2.1 cmH_2_O, *p* < 0.001), E_L_ (8.9 ± 1.9 vs. 29.9 ± 5.9 cmH_2_O/mL, *p* = 0.003), and E_CW_ (32.8 ± 3.2 vs. 4.4 ± 1.8 cmH_2_O/mL, *p* < 0.001), while EELV/kg, ΔP, C_RS_, and E_RS_ did not show significant differences (see Table 2 and Figure 2, Figure 3, Figure 4, Figure 5, Figure 6 and Figure 7).

Figure 2 illustrates the alterations of EELV in relation to bodyweight over six hours of ventilation in response to elevation of intraabdominal pressures (10 mmHg in groups A and C and 20 mmHg in group B, respectively) and induction of ARDS (group C).

Figure 3 illustrates the alterations of driving pressure over six hours of ventilation in response to elevation of intraabdominal pressures (10 mmHg in groups A and C and 20 mmHg in group B, respectively) and induction of ARDS (group C).

Figure 4 illustrates the alterations of transpulmonary pressure over six hours of ventilation in response to elevation of intraabdominal pressures (10 mmHg in groups A and C and 20 mmHg in group B, respectively) and induction of ARDS (group C).

Figure 5 illustrates the alterations of static compliance of the respiratory system over six hours of ventilation in response to elevation of intraabdominal pressures (10 mmHg in groups A and C and 20 mmHg in group B, respectively) and induction of ARDS (group C).

Figure 6 illustrates the alterations of static elastance of the lung over six hours of ventilation in response to elevation of intraabdominal pressures (10 mmHg in groups A and C and 20 mmHg in group B, respectively) and induction of ARDS (group C).

Figure 7 illustrates the alterations of static elastance of the chest wall over six hours of ventilation in response to elevation of intraabdominal pressures (10 mmHg in groups A and C and 20 mmHg in group B, respectively) and induction of ARDS (group C).

### 3.4. Oxygenation after 6 h

Alterations in P/F ratio (51 ± 18 vs. −213 ± 67 mmHg, *p* = 0.001) were significantly different between group B and C (see Table 2).

### 3.5. Hemodynamic Parameters after 6 h

We did not find differences in the alterations of hemodynamic parameters between the groups (see Table 2).

## 4. Discussion

In this porcine model of IAH and ARDS, we observed a reduction of EELV and elevated airway pressures in all groups six hours after increasing the IAP and inducing an ARDS, respectively. Although there were no significant differences between the IAH and ARDS groups in the alterations of global respiratory parameters (specifically ΔP, EELV/kg, C_RS_, and E_RS_), specific parameters of lung and chest wall mechanics showed significant differences (specifically E_L_, E_CW_, and TPP).

Our data showing elevated airway pressures and reduced EELV in pigs with ARDS and IAH are consistent with previous findings in both pigs and humans, suggesting generalizability [6,11,12,30]. Previous findings from our group have indicated that a PEEP of 10 cmH_2_O is best suitable for patients with moderately elevated intra-abdominal pressure of 10 mmHg, which is commonly seen in ICU patients [19,20]. Therefore, we used this setting of IAP = 10 mmHg and PEEP = 10 cmH_2_O as the reference group. Significant differences were observed among the groups at baseline. To account for this, we conducted statistical comparisons of the mean value alterations within the groups.

Our findings demonstrate that global respiratory parameters, such as ΔP and C_RS_, do not differ significantly between pigs with IAH or ARDS in a standardized experimental setup. Other studies exploring the effects of IAH or ARDS on respiratory mechanics separately have also shown impairment of global respiratory parameters in both conditions, in humans and in pigs alike [9,11,19,31,32,33]. Commonly accepted strategies for lung-protective ventilation focus on global parameters such as lower tidal volume (6 mL/kg predicted body weight) and limitation of P_peak_ (30 cmH_2_O) [34]. Furthermore, respiratory driving pressure has been found to be the best predictor of patient outcomes in ARF patients [31,35]. These findings underline the significance of global parameters easily and routinely measured with ICU ventilators. However, it is important to note that these global parameters are merely the sum of lung and chest wall mechanics. Thus, separate measurements of these compartments could be beneficial in understanding their individual contributions to the global impairment.

In contrast to global parameters, we found significant differences in the mechanics of the lung and chest wall between the IAH and ARDS groups, consistent with previous findings. For instance, Wauters et al. demonstrated that an elevation of IAP resulted in a reduction of C_RS_ due to an elevated E_CW_, while E_L_ remained unaffected [11]. Similarly, Gattinoni et al. also observed a significant correlation between elevated IAP and increased E_CW_ [36]. Additionally, they found that a decrease in C_RS_ in ARDS patients is primarily caused by an increase in E_L_, especially in non-aerated lung areas [37]. Notably, E_CW_ was only increased when ARDS occurred secondary to an underlying disease, such as polytrauma or peritonitis. These findings highlight that primarily intrapulmonary causes of ARF mainly affect lung mechanics, whereas primarily extrapulmonal causes tend to affect chest wall mechanics.

Our experiment demonstrates that stress (TPP) and strain (Vt/EELV) increased with elevation of IAP and induction of ARDS. Stress increased significantly more in the ARDS group than in the other groups, which can be explained by the excessive increase of E_L_ [38]. Strain was also substantially higher in the ARDS group, although not significantly due to high variance within the group. Therapeutic strategies of ARF, such as elevating PEEP, prone positioning, or abdominal decompression, primarily aim to reduce stress and strain on the lung parenchyma, in addition to treating the underlying disease. These parameters could indeed be considered the chief causes of VILI [5,38,39,40]. Further, from a physical standpoint, reducing stress and strain should mitigate VILI [31].

The substantially higher stress observed in the ARDS group demonstrates how small the energy-receiving lung area is in patients with ARDS compared to the IAH model, despite similar global airway pressures. Consequently, the potential for VILI is substantially higher in the ARDS lung, and global airway pressures may be inadequate surrogates for lung stress [38,40]. Therefore, separate measurements of lung and chest wall mechanics, using an esophageal probe, for example, are essential for proper evaluation of stress.

Our study had several limitations. Firstly, being an animal study, the findings may not be directly applicable to humans. Secondly, levels of PEEP and IAP were chosen arbitrarily, and the experimental phase lasted only six hours after artificially inducing IAH and ARDS, whereas clinical scenarios typically are not static and extend longer. Hence, clinical scenarios may reveal additional respiratory mechanics alterations. Thirdly, we did not strictly adhere to recommendations for lung-protective ventilation (ΔP, P_peak_, and V_t_). Consequently, the findings might differ from current clinical practices. Finally, significant baseline differences were observed among the groups. Although comparing the alterations within the groups attempted to mitigate this issue, it does not fully eliminate the impact of potential differences in the animals´ (patho-)physiology.

## 5. Conclusions

Global respiratory parameters indicate a severe impairment of respiratory mechanics six hours after artificially inducing IAH and ARDS in a porcine model. Although global airway pressures are the same in the IAH and ARDS groups, separate measurements of the lung and chest wall mechanics using an esophageal pressure probe reveal significant differences in the respiratory mechanics of the lung and chest wall between the groups. Therefore, separate measurements support the differentiation between primarily extra- and intrapulmonary problems and causes of ARF. Additionally, it helps revealing the real stress affecting the lung parenchyma during mechanical ventilation. It is important to note that this is an experimental animal study. Therefore, the results cannot be directly extrapolated to humans without limitations. We utilized fixed and static levels of PEEP and IAP, as well as an artificially induced model of ARDS. Consequently, it remains unclear how a more individualized matching of PEEP to IAP or PEEP to ARDS would have been tolerated and potentially beneficial. Clinical scenarios are also more heterogeneous and dynamic compared to our strict experimental protocol.

Given the substantial differences in treatment strategies based on etiology of ARF, physicians require tools to identify the most impaired compartment of the respiratory system to choose appropriate therapeutic strategies. Measuring the mechanics of the chest wall and lung separately could serve as one such tool and thereby improve patient care.

## Figures and Tables

**Table 1:** Baseline measurement. Table 1 shows the mean values ± SEM of respiratory and hemodynamic parameters of the different groups at baseline. Ventilator settings are equal for all groups: V_t_ 8 (mL/kg body weight), PEEP 5 (cmH_2_O), FiO_2_ 0.4, I:E 1:2.

	Group A (Mean Value ± SEM)	Group B (Mean Value ± SEM)	Group C (Mean Value ± SEM)	ANOVA (*p*-Value)
Body weight (kg)	49 ± 1	37 ± 1	49 ± 3	**<0.001**
IAP (mmHg)	1.8 ± 0.5	1.7 ± 0.6	2.1 ± 0.3	0.836
EELV/kg (mL/kg)	27 ± 3	30 ± 1	25 ± 1	0.249
P_peak_ (cmH_2_O)	16 ± 1	15 ± 1	17 ± 1	0.078
P_mean_ (cmH_2_O)	8.5 ± 0.2	8.3 ± 0.2	9.3 ± 0.3	**0.036**
Driving pres. (cmH_2_O)	11 ± 1	10 ± 1	12 ± 1	0.092
P_Esinsp_ (cmH_2_O)	12.2 ± 0.8	9.2 ± 1.0	9.8 ± 0.9	0.071
P_Esexp_ (cmH_2_O)	6.2 ± 0.3	4.3 ± 1.0	4.5 ± 0.7	0.166
TPP_insp_ (cmH_2_O)	4.0 ± 0.6	5.7 ± 1.0	7.3 ± 1.1	0.063
TPP_exp_ (cmH_2_O)	−1.2 ± 0.3	0.5 ± 1.0	0.5 ± 0.7	0.184
TPP (stress) (cmH_2_O)	5.2 ± 0.4	5.2 ± 0.4	6.8 ± 0.8	0.085
Strain (mL/mL)	0.31 ± 0.03	0.27 ± 0.01	0.33 ± 0.01	0.211
C_RS_ stat (mL/cmH_2_O)	42 ± 3	39 ± 4	40 ± 4	0.821
E_RS_ stat (cmH_2_O/mL)	29 ± 1	33 ± 2	32 ± 3	0.344
E_L_ stat (cmH_2_O/mL)	13 ± 1	17 ± 1	18 ± 2	0.165
E_CW_ stat (cmH_2_O/mL)	15 ± 2	16 ± 1	14 ± 1	0.532
Mech. power (J/min)	8.9 ± 0.3	6.3 ± 0.4	9.3 ± 0.5	**<0.001**
P/F ratio (mmHg)	425 ± 20	365 ± 15	453 ± 18	**0.009**
paCO_2_ (mmHg)	43 ± 2	42 ± 2	40 ± 2	0.615
Heart rate (bpm)	113 ± 9	77 ± 10	66 ± 7	**0.005**
MAP (mmHg)	98 ± 5	91 ± 1	89 ± 2	0.207
Lactate (mmol/L)	4.3 ± 1.0	1.2 ± 0.2	1.2 ± 0.1	**0.003**
Heart index (L/min/m^2^)	5.7 ± 0.3	4.1 ± 0.3	4.1 ± 0.3	**0.002**
GEDI (mL/m^2^)	651 ± 30	601 ± 29	664 ± 28	0.299
ELWI (mL/kg)	16 ± 1	13 ± 0	13 ± 1	0.105

bpm: beats per minute, cmH_2_O: centimeter of water column, C_RS_ stat: static compliance of the respiratory system, Driving pres.: driving pressure, E_CW_ stat: static elastance of the chest wall, ELWI: extravascular lung water, E_L_ stat: static elastance of the lung, E_RS_ stat: static elastance of the respiratory system, EELV: end-expiratory lung volume, FiO_2_: inspiratory oxygen fraction, GEDI: global end-diastolic volume index, I:E: ratio from inspiratory to expiratory time, IAP: intra-abdominal pressure, J/min; joule per minute, kg: kilogram, L/min/m^2^: liter per minute per square meter, MAP: middle arterial pressure, mech. power: mechanical power, mL: milliliter, mmHg: millimeter mercury, mmol/L: millimole per liter, P/F ratio: ratio between arterial pressure of oxygen and inspired oxygen concentration, paCO_2_: partial pressure of carbon dioxide, P_Esexp_: esophagus pressure at expiration, PEEP: positive end-expiratory pressure, P_Esinsp_: esophagus pressure at inspiration, P_mean_: mean airway pressure, P_peak_: peak airway pressure, SEM: standard error of the mean, TPP: transpulmonary pressure, TPP_exp_: transpulmonary pressure at expiration, TPP_insp_: transpulmonary pressure at inspiration, V_t_: tidal volume.

**Table 2:** Alteration measurement after 6 h. Table 2 shows the mean values ± SEM of the alterations of respiratory and hemodynamic parameters within the groups after 6 h to baseline (H6-H0). Ventilator settings equal for all groups during experimental phase: V_t_ 8 (mL/kg body weight), PEEP 10 (cmH_2_O), FiO_2_ 0.4, I:E 1:2.

	Group A (Mean Value ± SEM)	Group B (Mean Value ± SEM)	Group C (Mean Value ± SEM)	ANOVA (*p*-Value)	Post hoc A vs. B (*p*-Value)	Post hoc A vs. C (*p*-Value)	Post hoc B vs. C (*p*-Value)
ΔIAP (mmHg)	8.3 ± 0.4	18.3 ± 0.6	8.1 ± 0.4	<0.001	<0.001	1	<0.001
ΔEELV/kg (mL/kg)	−3.6 ± 0.7	−6.1 ± 1.3	−11.0 ± 2.5	0.022	0.939	0.022	0.171
ΔP_peak_ (cmH_2_O)	8.3 ± 0.6	17.7 ± 0.8	18.0 ± 2.3	<0.001	0.001	<0.001	1
ΔP_mean_ (cmH_2_O)	6.2 ± 0.3	9.5 ± 0.6	9.2 ± 1.2	0.018	0.029	0.053	1
ΔDriving pres. (cmH_2_O)	3.3 ± 0.6	12.5 ± 0.9	13.2 ± 2.3	<0.001	0.002	<0.001	1
ΔP_Esinsp_ (cmH_2_O)	7.3 ± 1.3	14.7 ± 1.3	7.8 ± 1.3	0.001	0.003	1	0.005
ΔP_Esexp_ (cmH_2_O)	5.5 ± 1.1	4.8 ± 0.9	6.0 ± 0.9	0.694	1	1	1
ΔTPP_insp_ (cmH_2_O)	1.0 ± 1.4	3.0 ± 0.7	10.2 ± 2.6	0.005	1	0.006	0.031
ΔTPP_exp_ (cmH_2_O)	−0.5 ± 1.1	0.3 ± 1.0	−1.2 ± 0.8	0.55	1	1	0.849
ΔTPP (stress) (cmH_2_O)	1.5 ± 0.5	2.7 ± 0.6	11.3 ± 2.1	<0.001	1	<0.001	<0.001
ΔStrain (mL/mL)	0.05 ± 0.01	0.07 ± 0.01	0.55 ± 0.31	0.115	1	0.198	0.232
ΔC_RS_ stat (mL/cmH_2_O)	−9.7 ± 2.7	−21.7 ± 2.8	−19.5 ± 3.4	0.027	0.036	0.1	1
ΔE_RS_ stat (cmH_2_O/mL)	8.5 ± 1.6	41.7 ± 3.1	34.3 ± 6.5	<0.001	<0.001	0.002	0.714
ΔE_L_ stat (cmH_2_O/mL)	3.8 ± 1.3	8.9 ± 1.9	29.9 ± 5.9	<0.001	1	<0.001	0.003
ΔE_CW_ stat (cmH_2_O/mL)	4.8 ± 1.9	32.8 ± 3.2	4.4 ± 1.8	<0.001	<0.001	1	<0.001
ΔMech. power (J/min)	5.6 ± 0.3	7.3 ± 0.3	9.5 ± 0.9	<0.001	0.153	<0.001	0.036
ΔP/F ratio (mmHg)	−29 ± 16	51 ± 18	−213 ± 67	0.001	0.563	0.02	0.001
ΔpaCO_2_ (mmHg)	−2.5 ± 2.3	2.5 ± 2.1	−1.1 ± 2.3	0.297	0.405	1	0.854
ΔHeart rate (bpm)	−14 ± 5	11 ± 11	18 ± 12	0.085	0.272	0.111	1
ΔMAP (mmHg)	4.2 ± 5.2	18.5 ± 3.4	10.2 ± 5.1	0.12	0.131	1	0.66
ΔLactate (mmol/L)	−3.2 ± 1.1	−0.6 ± 0.2	−0.5 ± 0.1	0.011	0.026	0.023	1
ΔHeart index (L/min/m^2^)	−0.9 ± 0.3	−0.3 ± 0.4	−0.1 ± 0.3	0.319	0.816	0.458	1
ΔGEDI (mL/m^2^)	−22 ± 19	−55 ± 24	39 ± 32	0.059	1	0.343	0.062
ΔELWI (mL/kg)	0.0 ± 0.7	0.5 ± 0.3	1.8 ± 1.4	0.38	1	0.555	0.985
Total crystalloids (L)	6.3 ± 0.5	5.4 ± 0.3	6.0 ± 0.6	0.391	0.549	1	1

Δ: delta from baseline to hour 6, bpm: beats per minute, cmH_2_O: centimeter of water column, C_RS_ stat: static compliance of the respiratory system, driving pres.: driving pressure, E_CW_ stat: static elastance of the chest wall, ELWI: extravascular lung water, E_L_ stat: static elastance of the lung, E_RS_ stat: static elastance of the respiratory system, EELV: end-expiratory lung volume, FiO_2_: inspiratory oxygen fraction, GEDI: global end-diastolic volume index, I:E: ratio from inspiratory to expiratory time, IAP: intra-abdominal pressure, J/min; joule per minute, kg: kilogram, L: liter, L/min/m^2^: liter per minute per square meter, MAP: middle arterial pressure, mech. power: mechanical power, mL: milliliter, mmHg: millimeter mercury, mmol/L: millimole per liter, P/F ratio: ratio between arterial pressure of oxygen and inspired oxygen concentration, paCO_2_: partial pressure of carbon dioxide, P_Esexp_: esophagus pressure at expiration, PEEP: positive end-expiratory pressure, P_Esinsp_: esophagus pressure at inspiration, P_mean_: mean airway pressure, P_peak_: peak airway pressure, SEM: standard error of the mean, TPP: transpulmonary pressure, TPP_exp_: transpulmonary pressure at expiration, TPP_insp_: transpulmonary pressure at inspiration, V_t_: tidal volume.

## Data Availability

The data presented in this study are partially available on request from the corresponding author due to ongoing research.
